# Supplementary material for: Perceptual rivalry with vibrotactile stimuli
Source: Atten Percept Psychophys. 2021 Apr 22;83(6):2613–24. doi: 10.3758/s13414-021-02278-1 (PMC8302565; doi:10.3758/s13414-021-02278-1)
Supplement: Supplementary file 1 — (PDF 754 KB) [file 13414_2021_2278_MOESM1_ESM.pdf]

Noname manuscript No.
(will be inserted by the editor)

Supplementary material

Farzaneh Darki · James Rankin

Received: date / Accepted: date

For Review Only

F. Darki
Department of Mathematics, College of Engineering, Mathematics & Physical Sciences, University of Exeter
E-mail: fd303@exeter.ac.uk

J. Rankin
Department of Mathematics, College of Engineering, Mathematics & Physical Sciences, University of Exeter

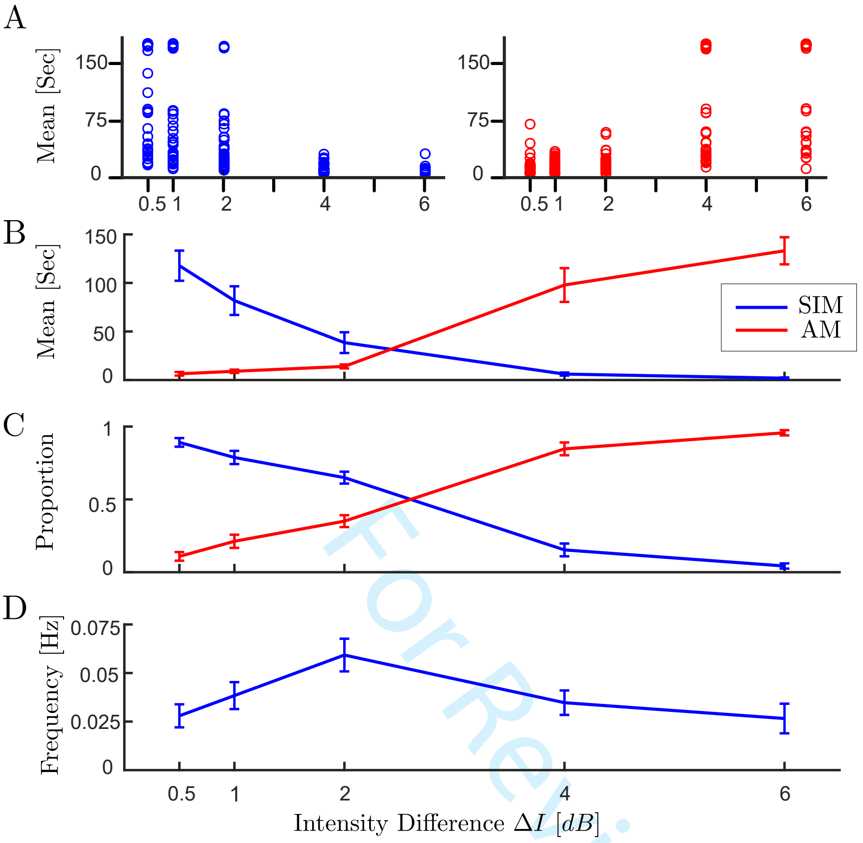

Fig. 1 Levelt's proposition II **(A)** The distribution of dominance durations at each trial with different experimental conditions (45 observation per experimental condition). **(B)** Mean dominance duration, **(C)** proportion of dominance for each percept type, **(D)** alternation rate, as a function of intensity difference (ΔI).

For Review Only

Table 1 Two-way repeated measure ANOVA of proportion of **both percept types (AM, SIM)** with respect to intensity difference (ΔI) and percept type. Analysis shows a significant effect of ΔI:percept on the proportion.

Source	df_{num}	df_{den}	F	p	ges	$p[GG]$
Percept	1	41	0.09	.76	.001	
ΔI	4	164	0.91	.46	.01	
ΔI:Percept	4	164	201.85	$< .001$	.71	$< .001$

Table 2 One-way repeated measure ANOVA of proportion of **SIM** perception with respect to intensity difference (ΔI). Analysis shows a significant effect of the intensity difference on the proportion.

Source	df_{num}	df_{den}	F	p	ges	$p[GG]$
ΔI	4	164	114.01	$< .001$	.07	$< .001$

Table 5 Pairwise ttest, with Bonferoni corrected p-values, on the proportions of **AM** perception with respect to intensity difference (ΔI).

	$\Delta I = 0.5$	$\Delta I = 1$	$\Delta I = 2$	$\Delta I = 4$
$\Delta I = 1$	.08	-	-	-
$\Delta I = 2$	.01	1.00	-	-
$\Delta I = 4$	$< .001$	$< .001$	$< .001$	-
$\Delta I = 6$	$< .001$	$< .001$	$< .001$	$< .001$

Table 6 One-way repeated measure ANOVA of frequency of **both percept types (AM, SIM)** with respect to intensity difference (ΔI). Analysis shows a significant effect of ΔI on the frequency.

Source	df_{num}	df_{den}	F	p	ges	$p[GG]$
ΔI	4	176	15.98	$< .001$	.12	$< .001$

Table 7 Pairwise ttest, with Bonferoni corrected p-values, on the frequency of **both percept types (AM, SIM)** with respect to intensity difference (ΔI).

	$\Delta I = 0.5$	$\Delta I = 1$	$\Delta I = 2$	$\Delta I = 4$
$\Delta I = 1$	1.00	-	-	-
$\Delta I = 2$	$< .001$	.02	-	-
$\Delta I = 4$	1.00	1.00	.003	-
$\Delta I = 6$	.82	.82	$< .001$	1.00

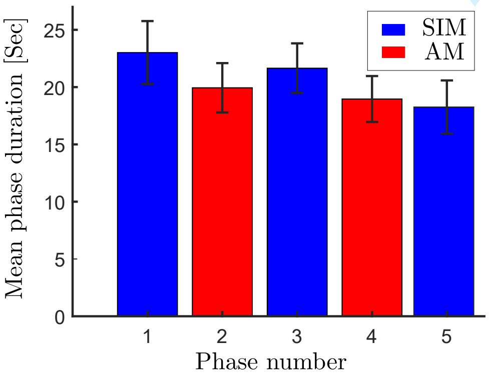

Fig. 3 Comparing the first five perceptual phases. As the result of one-way ANOVA analysis shows (Table 8), there is no significant difference between means of consecutive phase durations. Durations were averaged across all the trials in which participants had experienced five or more successive phases ($N = 106$).

Table 8 One-way repeated measure ANOVA of the first five phase with respect to phase number. Analysis shows there is no significant effect of phase number on the duration of phases.

Source	df_{num}	df_{den}	F	p	ges	$p[GG]$
phase	4	420	.71	.59	.005	.58

For Review Only

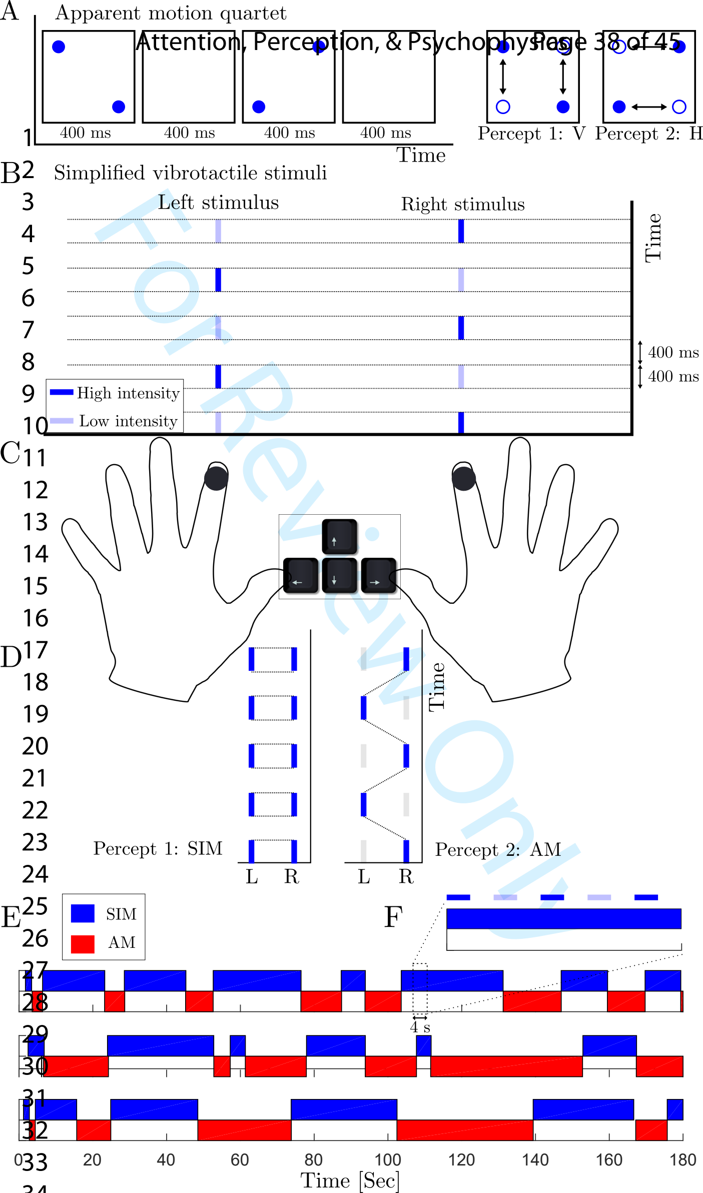

For Review Only

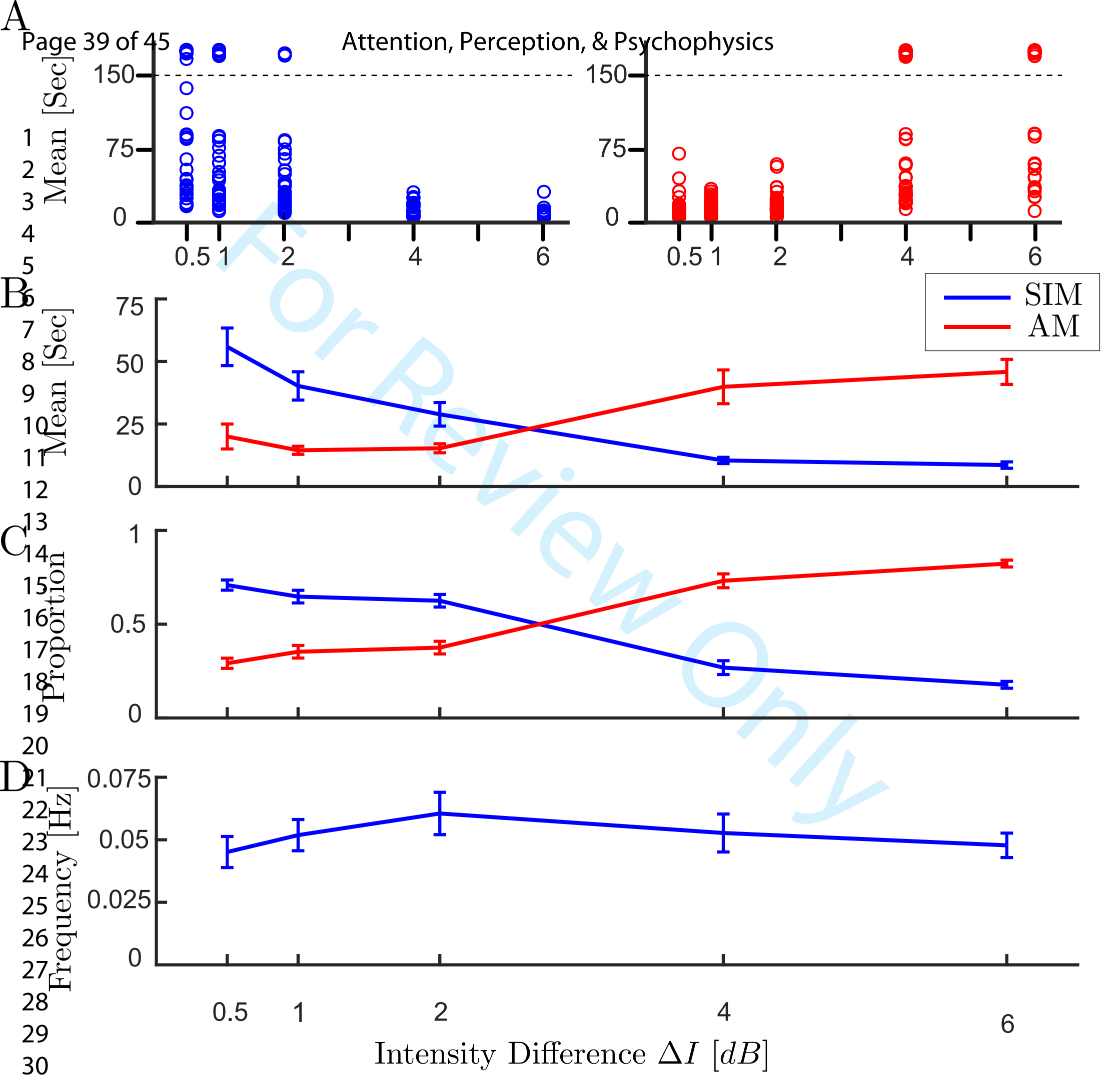

For Review Only

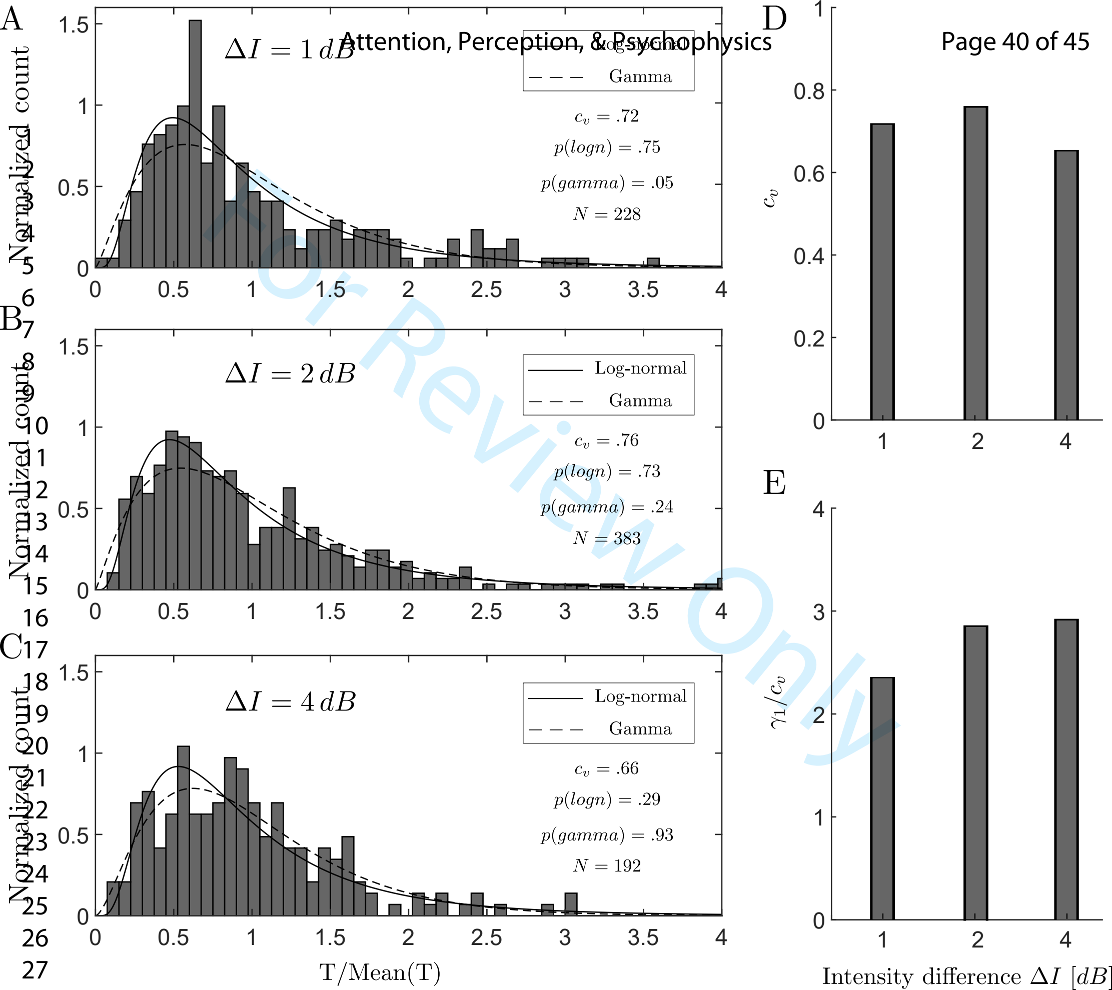

For Review Only

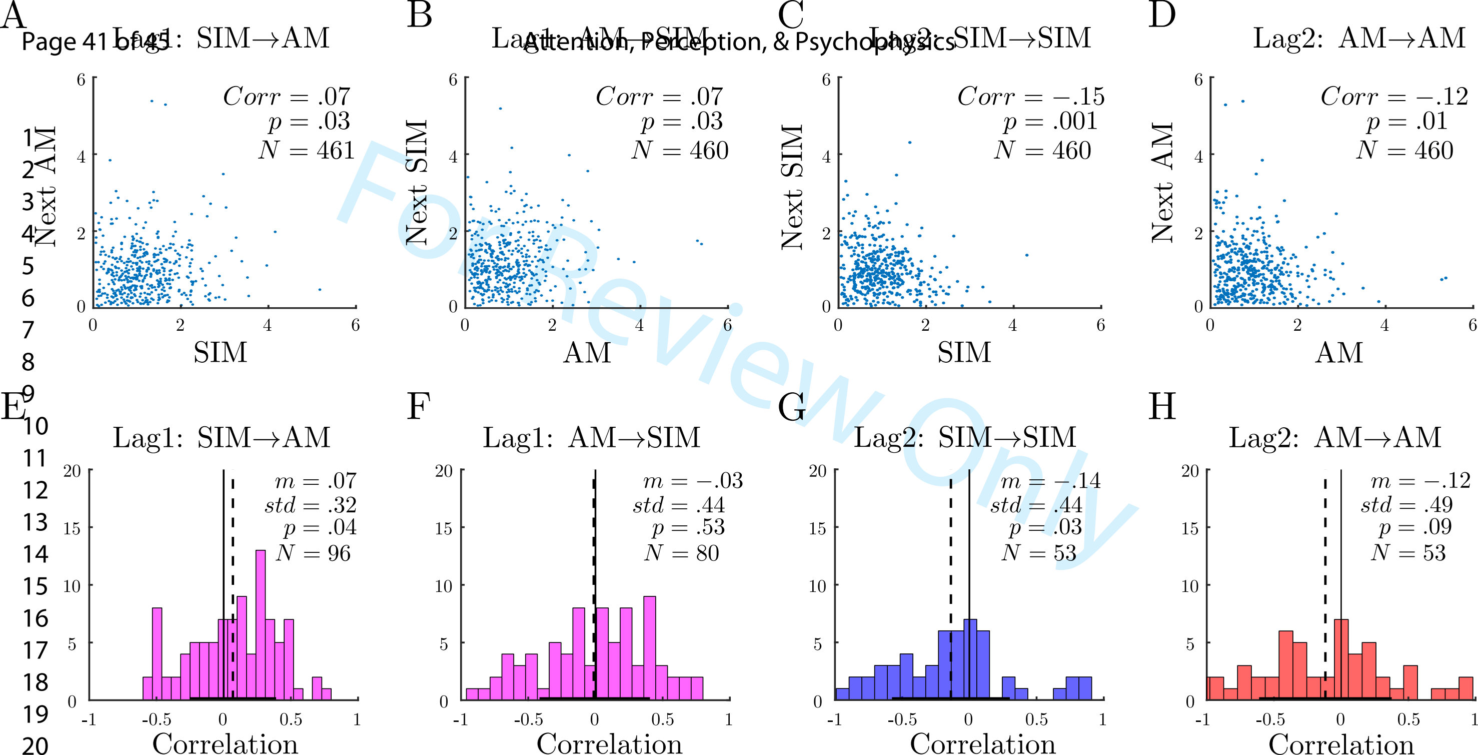

For Review Only

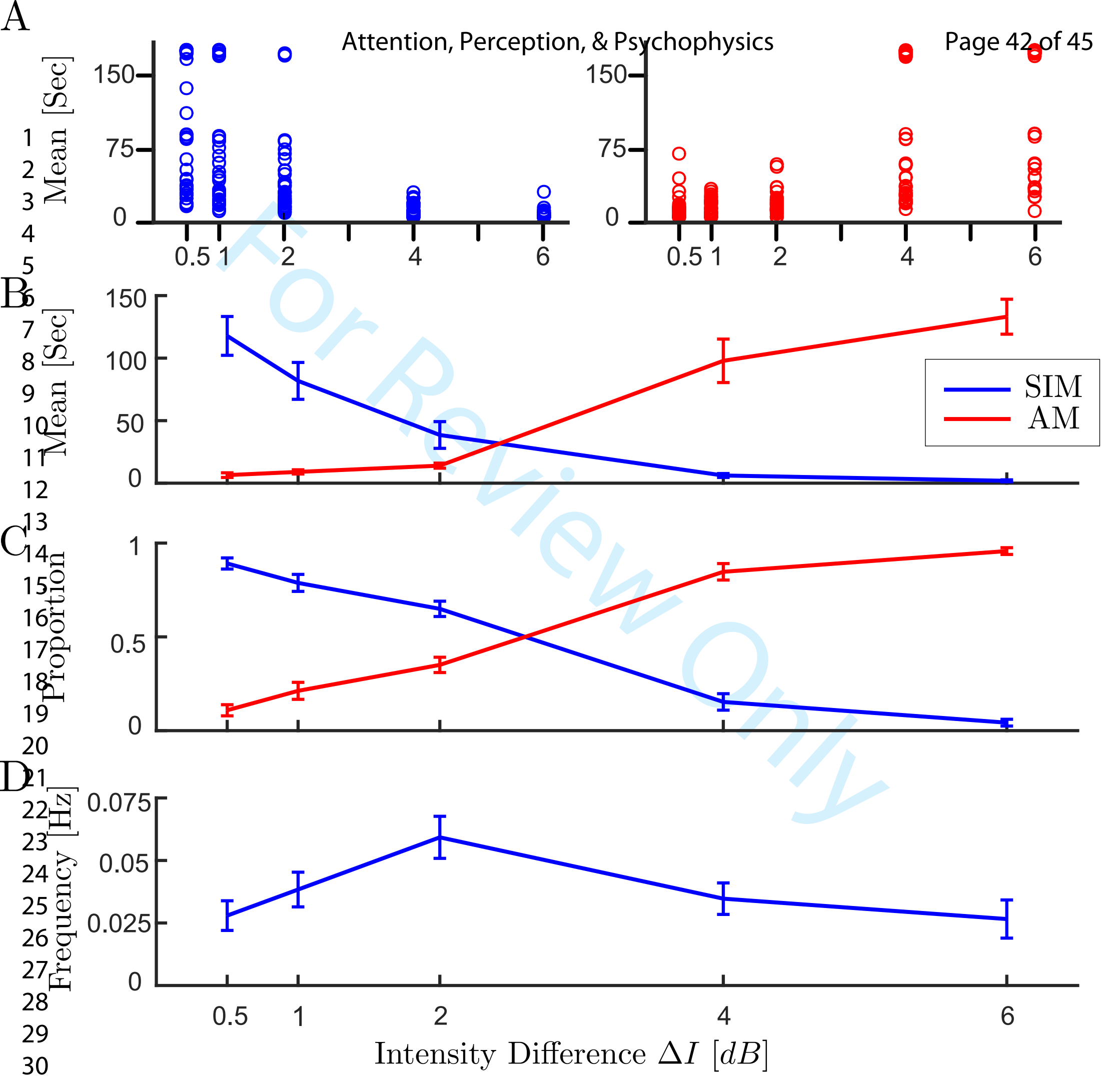

For Review Only

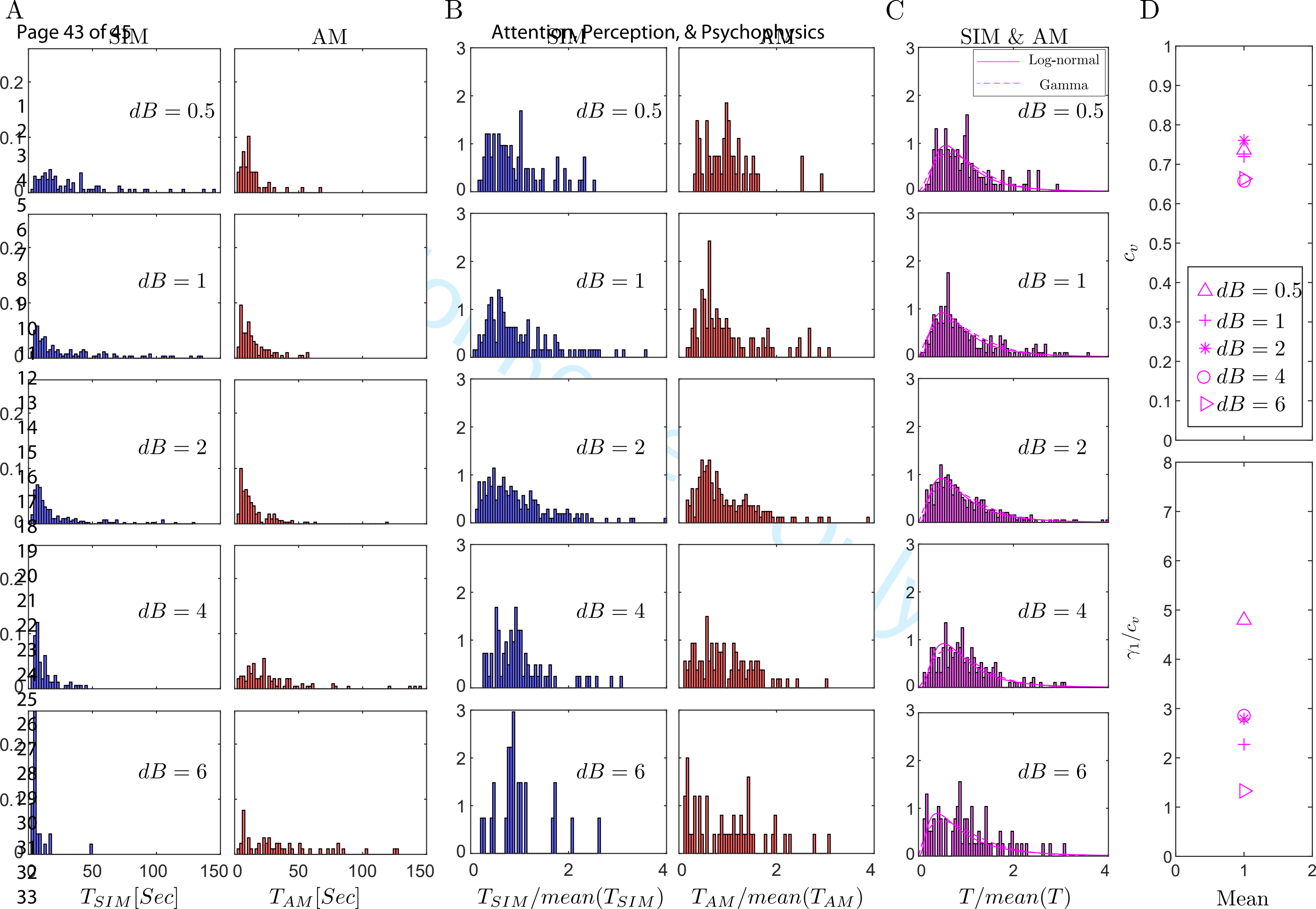
A
B
C
D
SIM
AM
SIM
AM
SIM & AM
Log-normal
Gamma
$dB = 0.5$
$dB = 1$
$dB = 2$
$dB = 4$
$dB = 6$
$T_{SIM}[Sec]$
$T_{AM}[Sec]$
$T_{SIM}/mean(T_{SIM})$
$T_{AM}/mean(T_{AM})$
$T/mean(T)$
c_v
γ_1/c_v
Mean

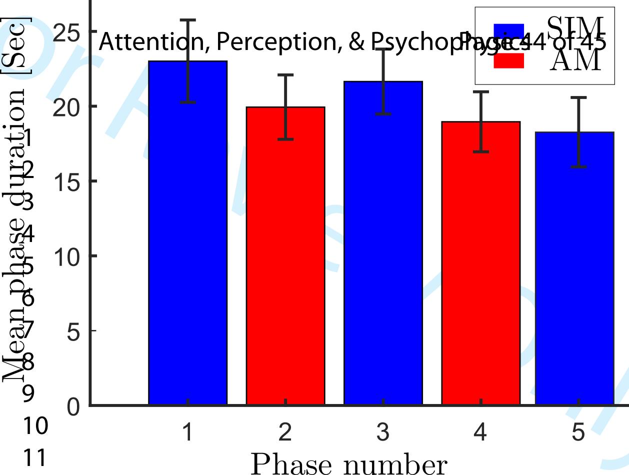
SIM
AM
Mean phase duration [Sec]
0
5
10
15
20
25
1
2
3
4
5
Phase number
